# Responsiveness and clinically important differences of the Western Ontario Rotator Cuff (WORC) Index in surgical and non-surgical treatment groups with different follow-up periods: A systematic review and meta-analysis

**DOI:** 10.1177/17585732241268631

**Published:** 2024-08-01

**Authors:** Maryam Farzad, Hassan Jafari, Joy C MacDermid, Milad Ataeian

**Affiliations:** 1 Hand and Upper Limb Center, St Joseph's Health Center, School of Physical Therapy, Department of Health and Rehabilitation Sciences, 6221 University of Western Ontario, London, Ontario, Canada; 2 School of Occupational Therapy, the University of Social Welfare and Rehabilitation Sciences, Tehran, Iran; 3 Department of Biostatistics & Health Informatics, Institute of Psychiatry, Psychology& Neuroscience, 4616 King's College London, London, UK; 4 Physical Therapy and Surgery, Western University, London, Ontario, Canada; 5 Clinical Research Lab, Hand and Upper Limb Centre, St Joseph's Health Centre, London, Ontario, Canada; 6 School of Physical Therapy, Department of Health and Rehabilitation Sciences, 6221 University of Western Ontario, London, Ontario, Canada

**Keywords:** Western Ontario Rotator Cuff Index, minimum clinically important difference, patient-reported outcome measures, orthopedic research, systematic review, meta-analysis

## Abstract

**Background:**

This study reviews and meta-analyzes the responsiveness and minimal clinically important difference (MCID) of the Western Ontario Rotator Cuff (WORC) Index for various patient populations and treatment durations.

**Methods:**

A comprehensive search in PubMed, Embase, Web of Science, and CINAHL identified studies on the responsiveness or MCID of the WORC in shoulder conditions. Two authors independently screened articles. Study quality was appraised using COSMIN and GRADE guidelines. Responsiveness was evaluated using anchor-based MCID and distribution-based standardized mean differences (SMDs) computed with Hedges’ *g*. A random-effect model addressed study variability, and heterogeneity was assessed with the chi-squared test and *I*² statistic.

**Results:**

The 12 studies yielded high-quality evidence supporting the WORC's responsiveness. A meta-analysis of 1326 observations revealed a significant overall effect size (SMD) of 0.91 (95% CI: 0.56 to 1.26; *p* < 0.0001), with high heterogeneity (*I*² = 91.2%). Subgroup analyses showed larger effect sizes for long-term follow-ups (SMD = 1.28) and surgical treatments (SMD = 1.14). The average MCID was 17 for conservative treatments within six months, 26 for surgical procedures, and 29 for follow-ups over six months.

**Conclusion:**

The WORC measures improvements in rotator cuff conditions, with varying MCID values for different treatments and durations.

## Introduction

Patient-reported outcome measures (PROMs) are essential tools for assessing the effectiveness of treatments and interventions in healthcare.^1^ Evaluating patient-reported outcomes, including quality of life (QOL), is crucial for assessing the effectiveness of treatments and interventions in shoulder conditions.^2^ The Western Ontario Rotator Cuff (WORC) is a well-established PROM in orthopedic research that evaluates the impact of shoulder conditions on the patient's daily life.^3^ This questionnaire assesses physical symptoms, sports and recreation, work, social function, and emotions. This tool helps clinicians identify areas of improvement, track progress, and make informed clinical decisions.^3,4^ Considering the significant impact of shoulder conditions on patients’ physical and mental well-being, incorporating PROMs into clinical practice is essential for delivering optimal patient care.

A critical aspect of WORC is the variability in its minimal clinically important difference (MCID)^5^ values, which are observed to differ significantly across diverse clinical settings and patient populations. Evaluating responsiveness and the minimum clinically important difference^5^ of PROM is essential to assess the effectiveness of treatments and interventions in healthcare.^5,6^ Responsiveness refers to the ability of a PROM to detect meaningful changes over time or following an intervention.^7^ The minimum clinically important difference^5^ is the smallest difference in a PROM that is considered clinically meaningful. Understanding the MCID provides a reference point for determining treatment success, identifying the clinical significance of responses, and comparing results across different treatments.^8^

While a score greater than the MCID signifies a minimum threshold for change, it does not necessarily mean the treatment is ‘successful’ from the patient's perspective. Research indicates that patients frequently regard a substantially larger improvement as indicative of success. Therefore, while the MCID provides a useful benchmark for clinical improvement, it does not alone determine the effectiveness of a treatment. Inferences about treatment effectiveness and causality should be made through randomized controlled trials, where treatments can be directly compared within the same patient population.^9,10^

There are different methods for evaluating responsiveness and MCID in PROMs, including anchor-based (external criterion) and distribution-based (internal criterion) methods.^11^ Anchor-based methods involve comparing PROM change scores with an external criterion, such as a reference standard or global rating of change scale. This method includes evaluating change against the selected reference anchor, receiver operating characteristic curve analysis, and area under the curve analysis. Distribution-based methods use statistical properties of the outcome distribution to establish the MCID and include the standard error of measurement (SEM), effect size, minimal detectable change, and the half-standard deviation (SD) method.^12,13^

Various factors are expected to impact MCID, including the responsiveness method used, the clinical population, the time interval over which change is assessed, and the intervention delivered, highlighting the need to evaluate MCID values across patient groups, time points, and interventions.^5^ Understanding these differences can help clinicians and researchers tailor patient treatment plans, ensuring that care is personalized, effective, and efficient.^12^ The availability of reliable MCID values can facilitate the interpretation of PROMs, minimize variability in outcomes, and improve the overall quality of care.^14^

This systematic review and meta-analysis evaluated the responsiveness and MCID critical values of the WORC questionnaire across various time points and interventions. Additionally, we conducted a meta-analysis to report the overall effect size, thereby quantifying the overall score change of the WORC before and after surgical and nonsurgical treatment.

## Methods

In February 2023, we registered this systematic review with the International Prospective Register of Systematic Reviews (PROSPERO) under the registration number CRD42023394542. We adhered to the Diagnostic Test Accuracy extension of the Preferred Reporting Items for Systematic Reviews and Meta-Analyses (PRISMA) guidelines.^15^ Moreover, we use the updated COSMIN reporting method in our reporting and drafting of the manuscript.^16^ A flowchart illustrates the study selection process, and a narrative summary of the included studies is provided. Furthermore, a meta-analysis is reported with appropriate measures of heterogeneity and a forest plot. A summary table is used to show the risk of bias assessment.

### Measure

The WORC questionnaire assesses the QOL in individuals with rotator cuff disorders.^17^ This questionnaire has 21 items and assesses five domains related to rotator cuff injuries, including physical symptoms, sports and recreation, work, lifestyle, and emotions. The content validity of the WORC is strong, as it covers a wide range of factors relevant to shoulder disorders.^3^

### Search strategy

A comprehensive electronic search was conducted on PubMed, Embase, Web of Science, CINAHL, and Google Scholar. The search included published studies up to the current date and used a combination of WORC with any of the following terms: ‘minimum clinically important difference’,^5^ ‘minimal important change’, ‘clinically important change’, ‘responsiveness’, and ‘sensitivity to change’. The search strategy was developed using appropriate Medical Subject Headings terms and combined keywords

### Eligibility criteria

Studies were included if they evaluated the responsiveness by any method and reported the MCID for the WORC in adult patients with shoulder conditions. Specifically, studies were eligible if they reported at least one indicator of MCID or responsiveness. Additionally, studies that compared the MCID with other outcome measures and provided details on the time frame, population, and intervention were included.

Exclusion criteria encompassed studies that did not report on the MCID of the WORC or only addressed the psychometric properties of these scales. Case reports, editorials, and conference proceedings were also excluded.

### Data extraction and charting

Two reviewers, MF and MA, independently screened all studies identified by the search strategy. The studies deemed relevant were assessed based on their titles and abstracts. The full-text articles of potentially relevant studies were retrieved for detailed review. Any disagreements with the reviewer were resolved through discussion with the senior author (JM) and consensus. Additionally, the reference lists of these studies were manually searched to identify other potentially eligible studies.

Information was extracted from all studies included in this review, comprising study design, population characteristics (age, sex, disease specifics, setting, country, and language version of the PROM), intervention details, and specific methods of calculating responsiveness and related values. These included the effect size, standardized response means (SRMs), clinically important differences, and/or MCID. The methods used for calculating MCID encompassed MCID estimation-based, anchor-based, or distribution-based approaches, all with 95% confidence intervals. We also extracted indicators of MCID or responsiveness, comparisons of MCID with other outcome measures, details on the study time frame, and descriptions of the interventions applied.

We calculated these values using the standard formula when studies did not specify the effect size^18^ and SRM. The effect size was calculated by dividing the mean difference by the SD of the baseline measurement. The SRM was calculated by dividing the difference between two measurement points with the SD for the difference within a group. We used the Cohen's benchmark to interpret the effect sizes observed in our study and assist clinical decision-making, including trivial (<0.20), small (≥0.20 to <0.50), moderate (≥ 0.50 to <0.80), or large (≥0.80).^2^

To address the challenge of obtaining a reliable anchor discriminating between ‘no change’ and ‘little improved’, we set the cutoff between ‘ improved’ and ‘non-improved.^19,20^’ We reviewed studies that used the global rating of changed (GRC) scale or similar measures to categorize improvement levels. Data on the number of patients categorized as ‘completely recovered’ or ‘much improved’ (improved) and those categorized as ‘slightly improved’, ‘unchanged’, ‘slightly worse’, ‘much worse’, or ‘worse than ever’ (non-improved) were extracted. Based on this data, patients were classified into ‘improved’ and ‘non-improved’ categories. The percentage of patients in each category for each study was then calculated.

Data were synthesized qualitatively and quantitatively to determine relationships between the MCID values and the population, intervention, or time frame.

### Quality assessment measurement quality and risk of bias

#### The methodological quality of each single study on a measurement property

The COSMIN risk of bias checklist,^21^ developed based on international consensus, was used to evaluate the methodological quality of each included study.^18^ The COSMIN risk of bias checklist rates the quality of studies in terms of nine measurement properties (content validity, structural validity, hypothesis testing, cross-cultural validity, criterion validity, internal consistency, reliability, measurement error, and responsiveness).^22^ Since the focus of this study is on responsiveness, only the items related to responsiveness were completed. The total score was determined by taking the lowest rating of the responsiveness items.^21^

COSMIN has identified four techniques for measuring responsiveness that are acceptable: a criterion approach, in which the instrument is compared with a gold standard or global rating scale that measures the same construct as the construct of interest, as well as three construct approaches, in which hypotheses are tested using comparisons with other outcome measures, comparisons between subgroups, and comparisons before and after an intervention, respectively. Area under curve (AUC)^23^ values of at least 0.70 are considered sufficient for distinguishing between participants who have improved and those who have not improved. The third and fourth methods, which include effect sizes (Cohen's *d* and SRMs) and paired *t*-tests, are typically not considered stand-alone methods for measuring responsiveness because they do not provide information about the validity of the change. However, they become acceptable when used in conjunction with hypothesis testing in a construct approach. In this context, paired *t*-tests are specifically used to assess the change in a particular outcome measure within the same group of participants over time. If these tests are integrated into a broader analytical framework that involves specific hypothesis testing, they are deemed appropriate for assessing responsiveness according to COSMIN standards.^24^

Two reviewers (MF and MA) completed the COSMIN risk of bias checklist, which included all the responsiveness items, with each reviewer indicating responsiveness on a five-point scale (‘very good’, ‘adequate’, ‘doubtful’, ‘inadequate’, or ‘not applicable’).

#### Criteria for good measurement properties

We used the COSMIN checklist to ensure that all essential measurement properties were considered in studies.^25^ The COSMIN checklist contains nine measurement properties that are clustered in three domains, ‘reliability’ (internal consistency, reliability, and measurement error), validity (content validity, structural validity, hypothesis testing, cross-cultural validity/measurement invariance, and criterion validity), and responsiveness.^22^ The COSMIN checklist was modified to only use the criteria for measuring the quality of the responsiveness. Two authors (MF and MA) independently assessed the included studies for methodological quality of responsiveness. Then, COSMIN criteria for good measurement properties were applied to rate the quality of measurement property of the responsiveness as sufficient (+), insufficient,^1^ or indeterminate.^12,26^ (Table 1)

**Table 1.** Criteria for good measurement properties.

Responsiveness	+	The result is in accordance with the hypothesis OR AUC ≥ 0.70
	?	No hypothesis defined (by the review team)
	-	The result is not in accordance with hypothesis OR AUC < 0.70

#### Qualitatively summarize the evidence and grade the quality of the evidence

The overall quality of each study on measuring responsiveness was rated by considering the lowest rating obtained in the checklist criteria. We pooled the consistent results and compared them against the criteria for good measurement properties to ascertain if the measurement property of the PROM is sufficient (+), insufficient (–), inconsistent (±), or indeterminate (?).

We utilized the Grading of Recommendations Assessment, Development, and Evaluation (GRADE) approach^27^ to evaluate the trustworthiness of our summarized findings. GRADE^28^ is a widely recognized method for appraising evidence quality in systematic reviews, particularly clinical trials. It aids in determining the confidence level we can place in the aggregate results of these trials. We adopted a modified GRADE approach to categorize the evidence quality as high, moderate, low, or very low (refer to Table 2). In this process, four key factors were considered: risk of bias (i.e. the methodological quality of the studies),^1^ inconsistency (i.e. unexplained inconsistency of results across studies),^6^ imprecision (i.e. total sample size of the available studies), and indirectness (i.e. evidence from populations different to the population of interest in the review). We assumed a starting point with the assumption that the pooled or overall result is high quality. The quality of evidence was subsequently downgraded by one or two levels when there was a risk of bias, (unexplained) inconsistency, imprecision (low sample size), or indirect results. The quality of evidence was also downgraded by three levels when the evidence was based on only one inadequate study (i.e. extremely serious risk of bias).

**Table 2.** Modified GRADE approach for grading evidence quality.

Quality level	Definitions
High	We are very confident that the true measurement property lies close to that of the estimate of the measurement property
Moderate	We are moderately confident in the measurement property estimate: the true measurement property is likely to be close to the estimate of the measurement property, but there is a possibility that it is substantially different
Low	Our confidence in the measurement property estimate is limited: the true measurement property may be substantially different from the estimate of the measurement property
Very low	We have very little confidence in the measurement property estimate: the true measurement property is likely to be substantially different from the estimate of the measurement property

#### Quantitatively pooled evidence or meta-analysis

Two separate measures of responsiveness were evaluated: distribution-based responsiveness using the effect size and anchor-based responsiveness using the MCID.^5^

*Anchor-based responsiveness with MCID*: In studies reporting the MCID for the WORC questionnaire, a mean and a range were extracted for two separate intervention types (surgical and conservative interventions) and two follow-up durations (less than six months and more than six months).

*Distribution-based responsiveness*: Effect size calculations were employed to measure responsiveness.^29^ The standardized mean differences (SMDs) were computed using Hedges’ *g* to quantify the effect size for postintervention differences between treatment and control groups over time.^30^ A 95% confidence interval (CI 95%) was computed for each effect size estimate. This approach allowed for the evaluation of responsiveness from a distribution-based perspective.

*Random-effects model*: A random-effect model was chosen for our meta-analysis to account for both within-study and between-study variability more rigorously. This approach is especially relevant due to the inherent heterogeneity in study designs, patient populations, and treatment protocols among the included studies. We used weighted means in our calculations that allowed us to obtain a more generalizable overall effect size based on the sample size.

*Heterogeneity assessment:* The degree of statistical heterogeneity among the included studies was quantitatively evaluated using the chi-squared (*χ*^2^) test and the *I*^2^ statistic.^31^ The *I*^2^ statistic serves as an estimator for the percentage of variance across studies that can be attributed to interstudy heterogeneity, as opposed to random error. A low *p*-value for *χ*^2^ indicates a significant heterogeneity in the estimated effect sizes. In terms of *I*^2^ values, thresholds of above 25%, 50%, and 75% suggest low, moderate, and high heterogeneity, respectively.

*Subgroup analyses*^32^: We conducted two distinct subgroup analyses. The first subgroup analysis summarized data from studies of patients who received conservative (nonsurgical) interventions. Follow-up durations were then categorized into short-term (less than six months) and long-term (more than six months) follow-ups.

*Sensitivity analysis*^33^*:* In the presence of heterogeneity, a sensitivity analysis was conducted by removing each study one at a time from the meta-analysis within each subgroup.

To enhance clinical relevance, we back-transformed the SMDs from the meta-analysis to their original scales by multiplying overall SMD by the pooled SD of the baseline measure, representing the actual magnitude of the effect in the original units.^34^ Additionally, we calculated the pooled SEM to assess the average precision of estimates and to distinguish true clinical changes from potential measurement errors. The SEM was first calculated by dividing the SD of mean differences by the square root of 2. We then compared these values to the mean MCID from the anchor-based responsiveness to explore if the overall score change met clinically relevant thresholds.

The meta-analysis was conducted using the ‘meta’ package, version 4.15–1, within the R Statistics software, version 4.0.2.

## Results

### Quality assessment measurement quality and risk of bias

Eight out of the twelve included studies (66.7%)^18,26,35–40^ demonstrated sufficient methodological quality with AUC values >0.70, indicating a sufficient level of rigor in assessing responsiveness. Two studies (16.7%)^41,42^ provided sufficient methodological quality based on their hypotheses, while one study (8.3%)^43^ showed insufficient methodological quality (Table 3).

**Table 3.** Characteristic of included studies.

Study	Language	Sample size	Diagnosis	Follow-up	Treatment	Mean age (SD)	Male/female	Pre	Post
(Wessel, Wolterbeek et al. 2018)		105	Arthroscopic rotator cuff repair (*n* = 37)	6m	Arthroscopic	57 ± 9.0 (41–75)	14/16	37.8 (15.3)	78.8 (15.0)
			Shoulder instability (*n* = 38)		Arthroscopic + PT + injection	54 ± 10.6 (24–72)	16/18	48.0 (24.8)	73.7 (23.5)
			Rotator cuff disorder		Arthroscopic + PT + Bankart	26 ± 9.4 (17–62)	21/5	55.7 (21.4)	89.8 (9.0)
									
(Baumgarten, Barthman et al. 2021)		50		6m-1Y	Arthroscopic rotator cuff repair			42 (17)	90 (14)
									
(St-Pierre, Dionne et al. 2015)	French-Canadian	87	Rotator cuff disorder	6w	Surgery	49.7 (12.4)	57/30	61.0 (24.2)	
									
(MacDermid, Drosdowech et al. 2006)		149	Rotator cuff disorder	6m	Surgery	55 (10.2)	96/53	147.5 (32.4)	72.7 (49)
									
(Braun and Handoll 2018)	German	65	Shoulder pain with partial-thickness rotator	3m	PT	50 (12)	40/25	89.7 (38)	53.4 (34.1)
									
(de Witte, Henseler et al. 2012)	Dutch	34	Rotator cuff tear, tendonitis, impingement	6w	PT, needling, and injection	55 (8.7)	43/49	46.8 (20.4)	61.7 (26.4)
									
(Brix, Bjørnholdt et al. 2020)	Danish	109	Impingement, biceps tendinitis and/or rotator cuff tears; were candidates for surgical treatment	3m	Surgery	55.4 (11.7)	52/57		
									
(Daghiani, Negahban et al. 2022)	Persian	130	Shoulder pain, including frozen shoulder, impingement syndrome, rotator cuff tear, tendinitis, and bursitis	4w	PT	46 (12.79)	56/74	31.70 (24.34)	
									
(Lopes, Ciconelli et al. 2009)	Brazil	30	Rotator cuff disorder	3m	PT/surgery	55.07 (10.83)	6/24	22.1 (22.18)	49.61 (32.78)
									
									
(Dewan, MacDermid et al. 2018)		223	Rotator cuff disorder	3m 6m	Repair (any technique)	56.7 (11.1)	151/72		
									
									
(Wang, Xie et al. 2017)	Chinese	124	Rotator cuff disorder	6m	Arthroscopic surgery	47.3 (9.5)	69/55	37.1 (13.4)	68.5 (19.0)
									
									
(Ekeberg, Bautz-Holter et al. 2010)	Norway	121	Rotator cuff disorder	6w	corticosteroid injection	51 (11)	44/77	1102.9 (331.4)	1161.4 (347)

Regarding measurement properties, four studies (33.3%)^26,37,38,40^ were rated as ‘Very Good’, indicating strong evidence for the responsiveness of the WORC. Conversely, five studies (58.3%)^18,35,36,41–44^ were rated as ‘Inadequate’, raising concerns about their responsiveness measurements (Table 4).

**Table 4.** COSMIN checklist scores evaluating methodology quality and measurement properties of each study for responsiveness.

	*N*	**Methodological quality**	**Result rating**	**Measurement properties**
(Wessel, Wolterbeek et al. 2018)	30	+	SRM:2.4	Inadequate
	34	+	SRM:1.01	Inadequate
	26	+	SRM:1.71	Inadequate
(Baumgarten, Barthman et al. 2021)	50	+	r = 0.90 (ASES)	Doubtful
(St-Pierre, Dionne et al. 2015)	87	+	SRM:1.54	Very good
(MacDermid, Drosdowech et al. 2006)	149	+	SRM:2	Inadequate
(Braun and Handoll 2018)	65	+		Inadequate
(de Witte, Henseler et al. 2012)	34	?	SRM: −0.91	Inadequate
(Brix, Bjørnholdt et al. 2020)	109	+	r = 0.71 (DASH)	Inadequate
(Daghiani, Negahban et al. 2022)	130	+	r = -0.79 (DASH)	Very good
(Lopes, Ciconelli et al. 2009)	30	+	SRM = 1.1	Inadequate
(Dewan, MacDermid et al. 2018)	223	+	SRM = 0.92	Very good
(Wang, Xie et al. 2017)	124	+	SRM = 1.52	Adequate
(Ekeberg, Bautz-Holter et al. 2010)	121	+	SRM = 1.69	Very good

The quality of evidence was graded using a modified GRADE approach. Considering no significant risk of bias, inconsistency, indirectness, or imprecision, the final quality of evidence for the responsiveness of the WORC measure was graded as ‘High’. This grading reflects a high confidence level in the measurement property estimates, with sufficient methodological quality supporting the responsiveness of WORC (Table 5).

**Table 5.** Grading the quality of evidence.

	Instrument	Risk of bias	Inconsistency	Indirectness	Imprecision	Final
**Responsiveness**	WORC	N0	0	0	0	High

### Study characteristics

**Figure 1.**
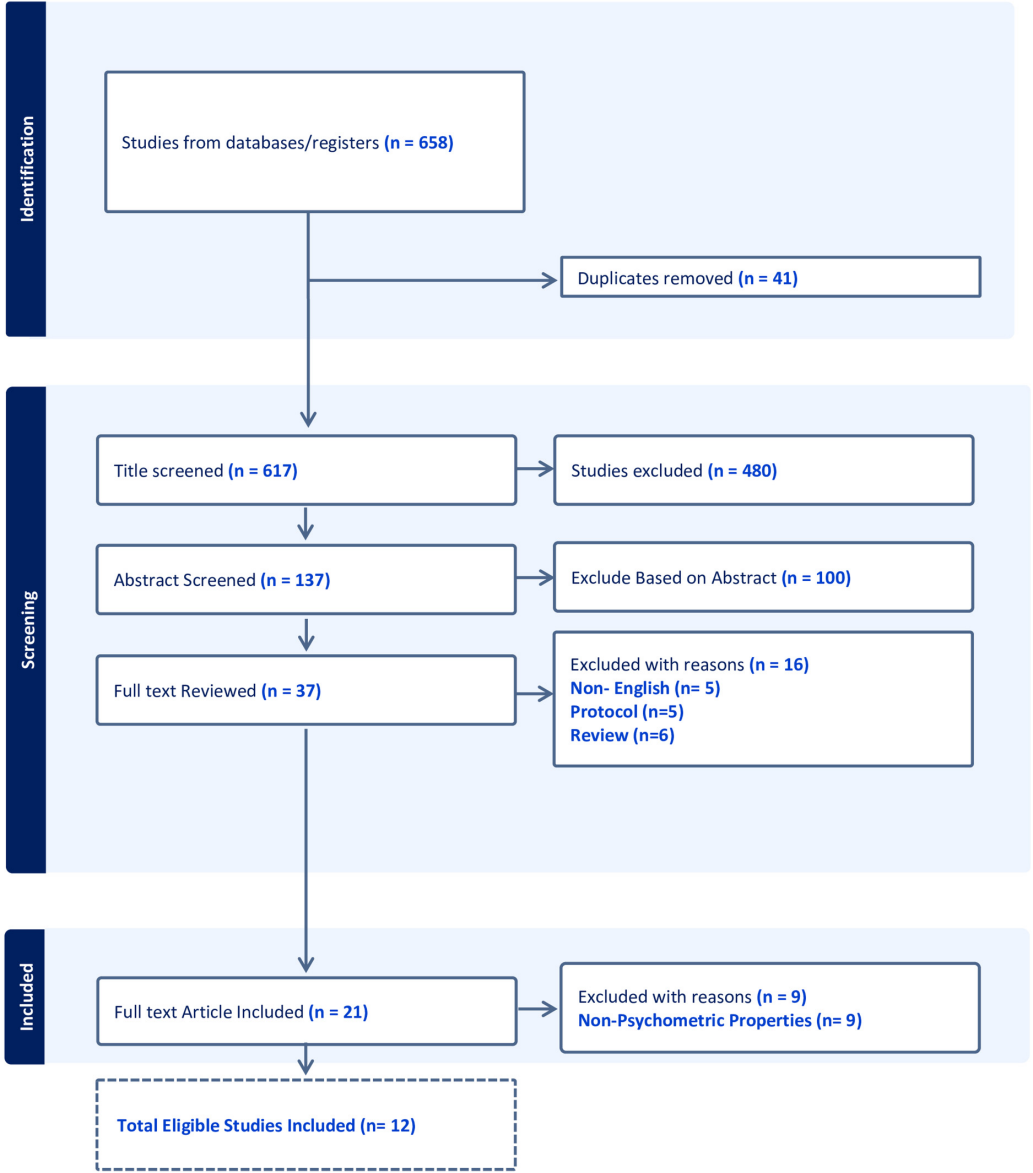
Flow diagram of study selection process. This figure illustrates the systematic review and meta-analysis screening procedure. Initially, 658 studies were identified from databases and registers. After removing 41 duplicates, 617 titles were screened, leading to the exclusion of 480 studies. The remaining 137 abstracts were reviewed, with 100 excluded based on the abstract content. A full-text review was conducted for 37 studies, out of which 16 were excluded for various reasons: non-English language (*n* = 5), protocol studies (*n* = 5), and review articles (*n* = 6). Ultimately, 21 full-text articles were included in the qualitative synthesis, with nine additional studies excluded for lacking psychometric properties, resulting in 12 studies meeting all eligibility criteria for inclusion.

In our systematic review, we initially included 12 studies (Figure 1). However, for analysis purposes, our final analysis involved 15 separate analyses. This modification was necessary because, in one study, the authors included three different methods of intervention and follow-up periods, which were treated as three separate studies.^38^ Seven studies (47%) reported WORC outcomes at short-term follow-ups,^26,36–38,42,43^ while eight (53%) reported at long-term follow-ups.^18,35,39,41,44^ Seven studies (47%) reported outcomes following surgical interventions,^18,35,36,38^ four studies (33.3%) after nonsurgical interventions,^26,38,42,43^ and three studies (20%) after both.^39,41,44^

Six studies (40%) employed anchor-based approaches with receiver operating characteristic analysis, using the GRC, disabilities of the arm, shoulder, and hand (DASH) score, and simple shoulder test (SST) as anchors. Five (41%) of the included studies used the GRC scale as an anchor. In these five studies, out of 280 patients, 155 patients (55.4%) were classified as improved, and 125 patients (44.6%) were classified as non-improved. Five studies (33.3%) used correlation methods, while the remaining four (26.7%) combined both approaches.

### Meta-analysis

A total of 12 studies comprising 1326 observations were included in the analysis. The mean MCID values were determined from the anchor-based responsiveness reported, were 15 (four studies) for conservative treatment within six-month follow-up, 20 (two studies) for surgical procedures under six-month follow-up, and 32.5 (two studies) for surgical procedures over six-month follow-up^18,35,37,43^ (Table 6).

**Table 6.** The values and the calculation methods for responsiveness.

Study	Follow-up	MCID calculation method	Cutoff method	Corresponding anchors and/or calculation methods	Candidate for surgery	MCID	SDC	SEM	SRM	ES
[^1^]	6m	Anchor	ROC	The shoulder hindrance score	Y	34	16.7	6	2.4	2.70
					Y	22.09	20.3	7.3	1.01	−0.42
					Y	31.08	25.4	9.1	1.71	
[^2^]	6m-1Y	Correlation		American Shoulder and Elbow Score	Y				2.3	
[^3^]	6w	Distributed Anchor	ROC	GRC	Y	17.5	12.3	5.3	1.54	1.32
[^4^]	6m	Correlation		Disabilities of the arm, shoulder, and hand (DASH) simple shoulder test [SST]	Y				2	
[^5^]	3m	Anchor	Regression	GRC	N	12				
[^6^]	6w	Correlation		DASH	N	11.7	19.1	6.9	0.91	-0.96
[^7^]	3m	Anchor Correlation		GRC	Y		21.20	7.73		
[^8^]	4w	Anchor	ROC	GRC	N	26.28	10.40	2.12		
[^9^]	3m	ES			N				1.1	0.92
[^10^]	3m	Correlation ES			Y				0.81	0.92
	6m								0.89	1.12
[^11^]	6m	ES			Y				1.69	1.92
[^12^]		Anchor	ROC	GRC	N	12	17.1	6.19	1.87	

MCID: minimal clinical important difference, SDC: smallest detectable change, SEM: standard error of measurement, SRM: standard response mean, ES: effect size, GRC: global rating of changes, ROC: receiver operation characteristic.

The pooled results from the random-effect model indicated a significant overall effect size (SMD) of 0.92, with a CI 95% ranging from 0.57 to 1.26, which is statistically significant (*z* = 5.11; *p* < 0.0001).

The positive effect sizes ranged from 0.24 to 2.65 for individual studies, with some showing more pronounced effect than others.

High levels of heterogeneity were observed among the included studies (*I*² = 91.2%; tau² = 0.4052), with a significant *Q*-value (*Q* = 147.44; df = 13, *p* < 0.0001) indicating variability beyond chance.

### Subgroup analyses

#### Follow-up duration

**Figure 2.**
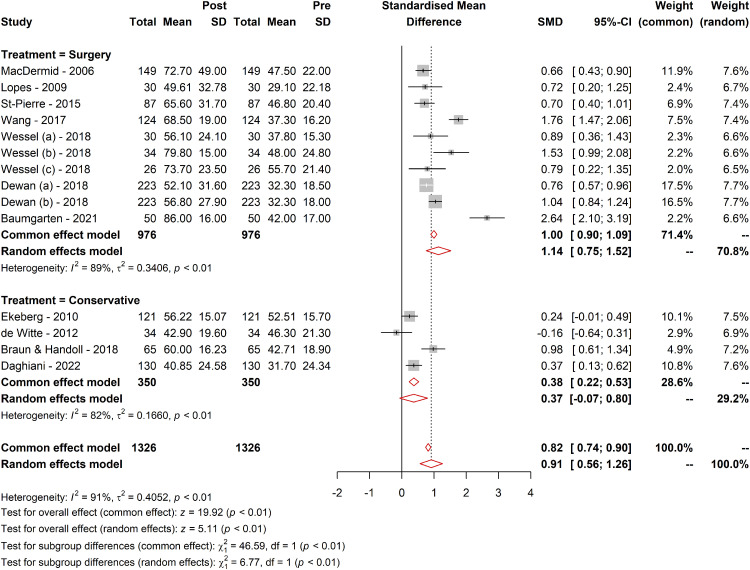
Responsiveness of WORC in different follow-up durations. Figure 2 presents a graphical representation of the responsiveness of the WORC Index across various follow-up durations. It illustrates the range of MCID values, SRMs, and other relevant metrics observed in different follow-up periods as identified in the systematic review. The data points in the figure show the variability in responsiveness, highlighting the differences between short-term (less than six months) and long-term (more than six months) follow-ups. The trend lines depict the general direction and magnitude of the responsiveness, providing a clear visual understanding of how the WORC's ability to detect clinically meaningful changes varies over time. This figure underscores the importance of considering follow-up duration in interpreting the WORC scores and their clinical implications.

The effect of follow-up duration on the WORC was explored through subgroup analyses. For the long-term follow-up subgroup, the effect size was larger (SMD = 1.28; 95% CI [0.74; 1.82]; tau² = 0.47) compared with the short-term follow-up subgroup (SMD = 0.58; 95% CI [0.28; 0.88]; tau² = 0.14). The test for subgroup differences confirmed that these differences were statistically significant in the random-effect model (*Q* = 4.96; df = 1, *p* = 0.02), suggesting that the treatment effect is larger over longer follow-up periods and WORC questionnaire responsive to detect these changes (Figure 2).

#### Treatment type

**Figure 3.**
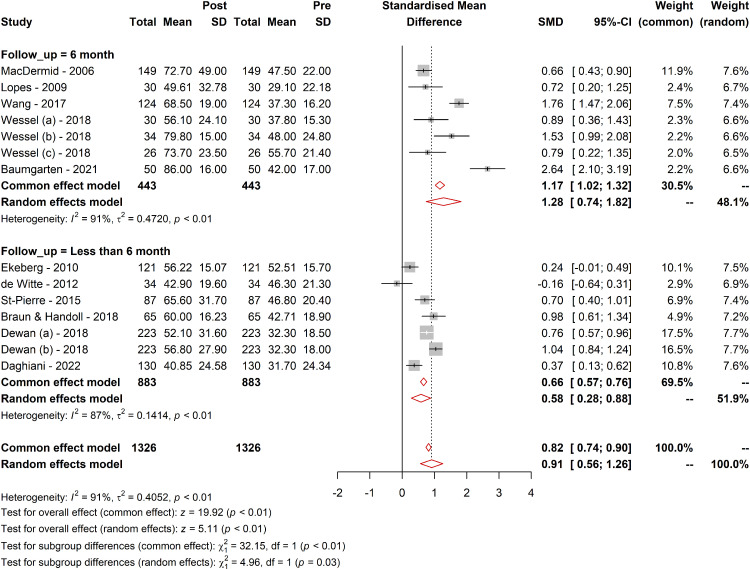
Comparative responsiveness of WORC across different treatment methods. Figure 3 visually represents the comparative responsiveness of the WORC Index across different treatment modalities. It displays the MCID values, SRMs, and other pertinent metrics for various treatment types, including surgical and conservative approaches. The figure highlights the variability in WORC responsiveness, delineating the differences in how effectively the WORC detects clinically meaningful changes in patients undergoing different types of treatments. The trends and data points in the figure provide a clear visual representation of the WORC's performance in diverse therapeutic contexts, emphasizing the critical role of the treatment type in interpreting WORC scores and their clinical significance.

Further subgroup analysis investigated the effect of the treatment type evaluated by the WORC. Patients undergoing surgery had a higher SMD (1.14; 95% CI [0.75; 1.52]; tau² = 0.34) compared to those receiving conservative treatment (SMD = 0.36; 95% CI [−0.06; 0.81]; tau² = 0.16). The subgroup difference was statistically significant (*Q* = 6.77; df = 1, *p* = 0.009), suggesting a distinct effect of the type of treatment, surgical comparing to nonsurgical measured by WORC (Figure 3).

#### Sensitivity analysis

A sensitivity analysis, which involved the sequential omission of individual studies from the meta-analysis, did not show any single study to disproportionately affect the overall effect size, indicating robustness in our meta-analysis findings.

### The clinical relevance

The meta-analysis revealed differences in effect sizes based on follow-up duration and intervention methods. For shorter-term effects, the overall effect sizes indicated that nonsurgical treatment patients experienced smaller but still noticeable improvements relatively quickly. The actual magnitude of the effect was 11.22, with a mean MCID of 17 and an SEM of 13.68. Conversely, for longer-term effects (more than six months), larger effect sizes were observed, particularly for patients receiving surgical interventions. The actual magnitude of the effect for longer-term follow-up was 25.34, with a mean MCID of 29 and an SEM of 14.00. For surgical interventions, the actual magnitude of the overall effect was 21.84, with a mean MCID of 26 and an SEM of 13.55. For nonsurgical treatments, the actual magnitude of the effect was 7.54, with a mean MCID of 16 and an SEM of 14.42. Considering the overall score changes for each subgroup, we see it often exceeded the related MCID for that subgroup. This indicates that observed changes were clinically relevant, especially in surgical and long-term follow-up subgroups. This suggests that a surgical treatment assessed after a six-month follow-up period might be more optimal for assessing clinically significant changes in rotator cuff tendinopathy patients.

## Discussion

Our systematic review and meta-analysis highlight the practical implications of the WORC questionnaire, a disease-specific QOL measure to evaluate symptoms and functional limitations associated with rotator cuff tendinopathy in daily practice.

The WORC questionnaire demonstrated a significant overall effect size, with larger effect sizes observed for long-term follow-ups and surgical treatments. Calculating the SEMs, compared with the mean MCID, indicates that observed score changes generally met clinically relevant thresholds, particularly in surgical and long-term follow-up subgroups.

Similar patterns were observed in the SMDs or the effect sizes^45^ from the provided forest plots. Baumgarten (2021)^35^ reported the highest effect (SMD = 2.65), while de Witte (2012)^43^ presented a negative effect (SMD = −0.16), indicating a slight decrease in the WORC score postintervention that was not statistically significant. Baumgarten's study, which involved surgical interventions with comprehensive follow-up, likely contributed to the substantial positive effect size, as surgical repairs enhance structural integrity and functional outcomes captured by the WORC. In contrast, de Witte's study, which involved less invasive ultrasound-guided interventions for calcific tendinitis, may not have produced immediate or significant improvements, resulting in the observed nonsignificant negative effect.

The variability of MCID across studies could be based on the varied methods used in each study^18^ For instance, one study used an anchor-based method, resulting in a relatively high MCID of 34, whereas another study using a correlation method with DASH and SST reported a lower MCID of 2.3. Anchor-based methods, which used the GRC as an anchor, directly tied MCID to clinically meaningful improvements from the patient's perception but are influenced by the subjective nature of the anchor.^7,46^ Conversely, while providing a more objective measure, correlation methods often produce lower MCID values and may not fully capture the clinical significance from the patient's viewpoint. A combined approach is recommended to ensure MCID is grounded in objective statistical rigor and subjective clinical significance, especially when various treatment methods and patient-reported outcomes exist.^7,47^

Despite not being part of our initial aim, the observed trends indicate that initial improvements with conservative treatments are significant. However, stronger effects and therefore bigger expected minimal threshold for clinical effects associated with surgical treatments in the long term highlight the sustained benefits of surgical approaches in managing rotator cuff injuries, especially when considering long-term patient−reported outcomes. Nonsurgical interventions showed more variability in improvement scores, suggesting lower effect; however due to the limited number of studies included in our meta-analysis for this subgroup, further research is recommended. Overall, for surgical and nonsurgical interventions, a follow-up duration in ranges within six months and longer is necessary for a more accurate and reliable assessment of MCID from a patient's perspective, leading to determine a more consistent and valid criterion for minimum score changes necessary for patient improvement.^48,49^

Additionally, social determinants of health (SDOH) such as gender, occupation, comorbidities, insurance status, and socioeconomic conditions significantly influence patient-reported outcomes, including QOL, after rotator cuff repairs. Therefore, it is crucial to consider each patient's circumstances, including their SDOH, to ensure equitable treatment access and optimize QOL outcomes.^45,50^

## Strengths and limitations of the study

Our study's strength lies in its comprehensive evaluation of the overall effect size, responsiveness, and MCID in the WORC scale across different treatment subgroups and follow-up intervals and comparing these indices in these subgroups. Additionally, the calculation of the SEM provided a deeper understanding of the precision and reliability of the WORC questionnaire. By offering these metrics, we have ensured that the observed changes in WORC scores are statistically significant and clinically meaningful. This detailed evaluation highlights the practical utility of the WORC questionnaire in assessing symptoms and functional limitations associated with rotator cuff tendinopathy, providing valuable insights for healthcare professionals to tailor treatment plans and accurately assess patient outcomes.

However, our study has several limitations. The potential heterogeneity introduced by the variability in methodologies across the included studies presents a challenge, as this variability could have influenced the measurement of the overall effect and the MCID values, potentially affecting the generalizability of our findings. Furthermore, the low number of studies, particularly those utilizing anchor-based methods, limits the robustness and reliability of our conclusions. A smaller number of anchor-based studies reduce the statistical power and precision of MCID estimates, potentially leading to broader confidence intervals and less definitive conclusions.^51^ The broad categorization of treatments into surgical or conservative groups may have impacted the precision of our findings, as this approach may overlook the specificities and nuances of different treatment modalities within these broad categories. Personal factors like age and sex were not adequately considered in the primary studies, which might influence the outcomes. Personal factors like age and sex were not adequately considered in the primary studies, which might influence the outcomes.

Future studies are not just a necessity but a crucial step toward advancing our understanding in this field. They are needed to evaluate responsiveness in different treatments more precisely. These studies should report statistics for clinically important subgroups, including health condition/severity, multiple follow-up time points, treatment options, and personal factors like age and sex. This requires larger samples to power such analyses. Responsiveness comparisons can only be confidently made through head-to-head comparisons. These studies should include comparison measures to help researchers and clinicians select the best option for specific measurements.
